# Noncompressive myelopathy in acute community‐acquired bacterial meningitis: Report of seven cases and review of literature

**DOI:** 10.1111/ene.16447

**Published:** 2024-09-02

**Authors:** Evelien H. G. M. Drost, Nora Chekrouni, Matthijs C. Brouwer, Diederik van de Beek

**Affiliations:** ^1^ Department of Neurology Amsterdam Neuroscience, Amsterdam University Medical Centres, University of Amsterdam Amsterdam the Netherlands

**Keywords:** bacterial meningitis, myelitis, spinal cord diseases

## Abstract

**Background and purpose:**

Bacterial meningitis is a severe disease with high rates of complications and unfavorable outcome. Complications involving the spinal cord are rarely reported.

**Methods:**

Cases of noncompressive myelopathy were identified from a nationwide cohort study of adults with community‐acquired bacterial meningitis in the Netherlands. The American Spinal Injury Association Impairment Scale was used to classify the severity of spinal cord dysfunction. Subsequently, we reviewed the literature on noncompressive myelopathy as a complication of bacterial meningitis.

**Results:**

Noncompressive myelopathy was reported in seven of 3047 episodes of community‐acquired bacterial meningitis (0.2%). The median age of these patients was 51 years (range = 17–77). Causative pathogens were *Streptococcus pneumoniae* in three, *Streptococcus agalactiae* in two, and *Neisseria meningitidis* and *Haemophilus influenzae* both in one. Paresis of legs (*n* = 6) or arms and legs (*n* = 1) was the presenting symptom, occurring after a median duration of 9 days after admission (range = 2–28). Spinal magnetic resonance imaging showed T2‐weighted abnormalities of the spinal cord in six of seven patients. Improvement of spinal cord function during admission was noted in four of seven patients. The literature review yielded 15 additional cases. Among patients from our cohort and the literature, there was no significant association between immunosuppressive therapy and subsequent improvement of spinal cord function (5/8 patients with immunosuppressive therapy [63%] vs. 5/14 without immunosuppressive therapy [36%], *p* = 0.44).

**Conclusions:**

Noncompressive myelopathy is an uncommon but severe complication of bacterial meningitis. Improvement after diagnosis is expected, but all patients had persistent neurological deficits.

## INTRODUCTION

Bacterial meningitis is a severe disease with high rates of intracranial complications and unfavorable outcome [1, 2]. Complications involving the spinal cord are extremely rare but reported to cause significant disability leading to poor outcome [3, 4, 5, 6, 7, 8, 9, 10, 11, 12, 13, 14, 15, 16]. Possible mechanisms of spinal cord dysfunction in bacterial meningitis include spinal ischemia due to vasculitis or circulatory shock, postinflammatory myelitis, and direct infection of the spinal cord [9, 15, 17]. Distinguishing these causes from spinal cord compression by abscess formation is essential in deciding the appropriate course of further treatment. Only a limited number of case reports of noncompressive myelopathy are available in literature, posing a challenge for clinicians to anticipate clinical progression and determine the appropriate treatment. We investigated the occurrence of noncompressive myelopathy complicating bacterial meningitis in a nationwide cohort study of patients with community‐acquired bacterial meningitis and reviewed the literature.

## METHODS

### Cohort

We included patients with community‐acquired bacterial meningitis in a nationwide prospective cohort study in the Netherlands between January 2006 and December 2023. Methods have been described previously [1]. All patients included in the cohort were 16 years of age or older and had a positive cerebrospinal fluid (CSF) culture or positive blood culture in combination with at least one individual CSF predictive factor for bacterial meningitis, defined as a glucose level of <1.9 mmol/L, a ratio of CSF glucose to blood glucose of <0.23, a protein level of >220 mg/dL, or a leukocyte count of >2000 cells/mm^3^ [18]. Patients were listed in the database of the Netherlands Reference Laboratory for Bacterial Meningitis (NRLBM), which receives approximately 90% of the isolates of all adult patients with bacterial meningitis. The NRLBM provided daily updates of the hospitals where the patients were admitted, and the patients' physicians were subsequently contacted. Physicians could also include patients not reported by the NRLBM. Written informed consent was obtained from all patients or their legally authorized representatives. The study was conducted in accordance with the Declaration of Helsinki and approved by the medical ethical review board of the Academic Medical Center, Amsterdam, the Netherlands.

From the cohort, we selected patients who experienced symptoms of spinal cord dysfunction secondary to bacterial meningitis. Spinal cord compression was ruled out by spinal magnetic resonance imaging (MRI) or spinal computed tomography (CT). The American Spinal Injury Association Impairment Scale (AIS) was used to classify the severity of the spinal cord dysfunction [19]. Grade A was defined as no remaining motor or sensory function below the site of injury, and grade E was attributed to a patient when there were normal motor and sensory function. Incomplete injuries were categorized into grades B, C, or D. Grade B was defined as the absence of motor function but retention of some sensory function. Grade C was characterized by the presence of some residual motor function, and grade D was defined as the preservation of functional motor ability, enabling movement against gravity. Patients were graded according to the AIS both during admission and at discharge. Improvement of spinal cord function was quantified as an advancement of at least one grade on the AIS during admission.

### Review of literature

We conducted a systematic search on PubMed database for articles in the English language published up to December 2023. Our search included the following terms: "bacterial meningitis," "spinal cord," and "myelopathy." Additional studies were identified by cross‐checking references. We combined these patients with the cases of the MeninGene cohort and described their clinical characteristics and outcome. We systematically scored clinical presentation, predisposing factors, ancillary investigation, AIS, and outcome. Outcome was graded at discharge according to the Glasgow Outcome Scale (GOS) [20]. This well‐validated measurement scale grades outcome from 1 to 5, in which 1 is death and 5 is defined as well recovery. Unfavorable outcome was defined as a score of 1–4. Differences between groups were compared using the chi‐squared test. Statistical analyses were performed using R statistics, version 4.0.3, and *p*‐values < 0.05 were considered significant.

## RESULTS

### Cohort

From January 2006 to December 2023, noncompressive myelopathy was reported in seven of 3047 (0.23%) episodes of community‐acquired bacterial meningitis included in our nationwide cohort study of adults with community‐acquired bacterial meningitis in the Netherlands (Table S1). Two of seven patients were female (29%), and the median age was 51 years (range = 17–77). None of the patients was immunocompromised. Four patients had predisposing factors for meningitis, consisting of either otitis or sinusitis in three patients and endocarditis in one patient. Fever was present in four of seven patients (57%), neck stiffness in five of seven (71%), and decreased level of consciousness in all patients. None of the patients exhibited neurological deficits suggestive of spinal cord injury upon admission. One patient presented with right‐sided hemiparesis and aphasia, raising suspicion of cerebral infarction, and another patient presented with right‐sided peripheral facial nerve palsy.

A lumbar puncture was performed in all patients. CSF culture revealed a positive result in six of seven patients. One patient had only a positive blood culture along with CSF predictive factors indicative of bacterial meningitis. Causative pathogens were *Streptococcus pneumoniae* in three patients, *Streptococcus agalactiae* in two patients, and *Neisseria meningitidis* and *Haemophilus influenzae* both in one patient. All patients received adequate antibiotic treatment from admission, and six patients were treated with adjunctive dexamethasone, all according to standard protocol (4 times 10 mg daily, for 4 days).

Symptoms of spinal cord dysfunction developed during admission after a median duration of 9 days (range = 2–28). In five patients, these symptoms coincided with the improvement of consciousness. The precise onset of spinal cord dysfunction and whether the clinical deterioration was acute was therefore unclear in these cases. Two patients developed spinal cord dysfunction after initial clinical improvement. Both patients experienced symptom progression over several days. Other focal neurological deficits were noted in five patients and consisted of cranial nerve palsies in four patients and hemiparesis and aphasia in one patient. In three patients, these symptoms manifested concurrently with spinal cord symptoms. Spinal cord dysfunction symptoms were paresis of the lower extremities in six patients and paresis of all four extremities in one patient. The AIS was used to classify the severity of the spinal cord dysfunction. One patient experienced complete spinal cord dysfunction below T8 and was graded as AIS grade A. The other four patients experienced incomplete spinal cord injury, graded as AIS grade B in two patients, AIS grade C in three patients, and AIS grade D in one patient. In five of seven patients, symptoms of bladder and bowel dysfunction were present; in the other two, it was unknown.

**FIGURE 1:**
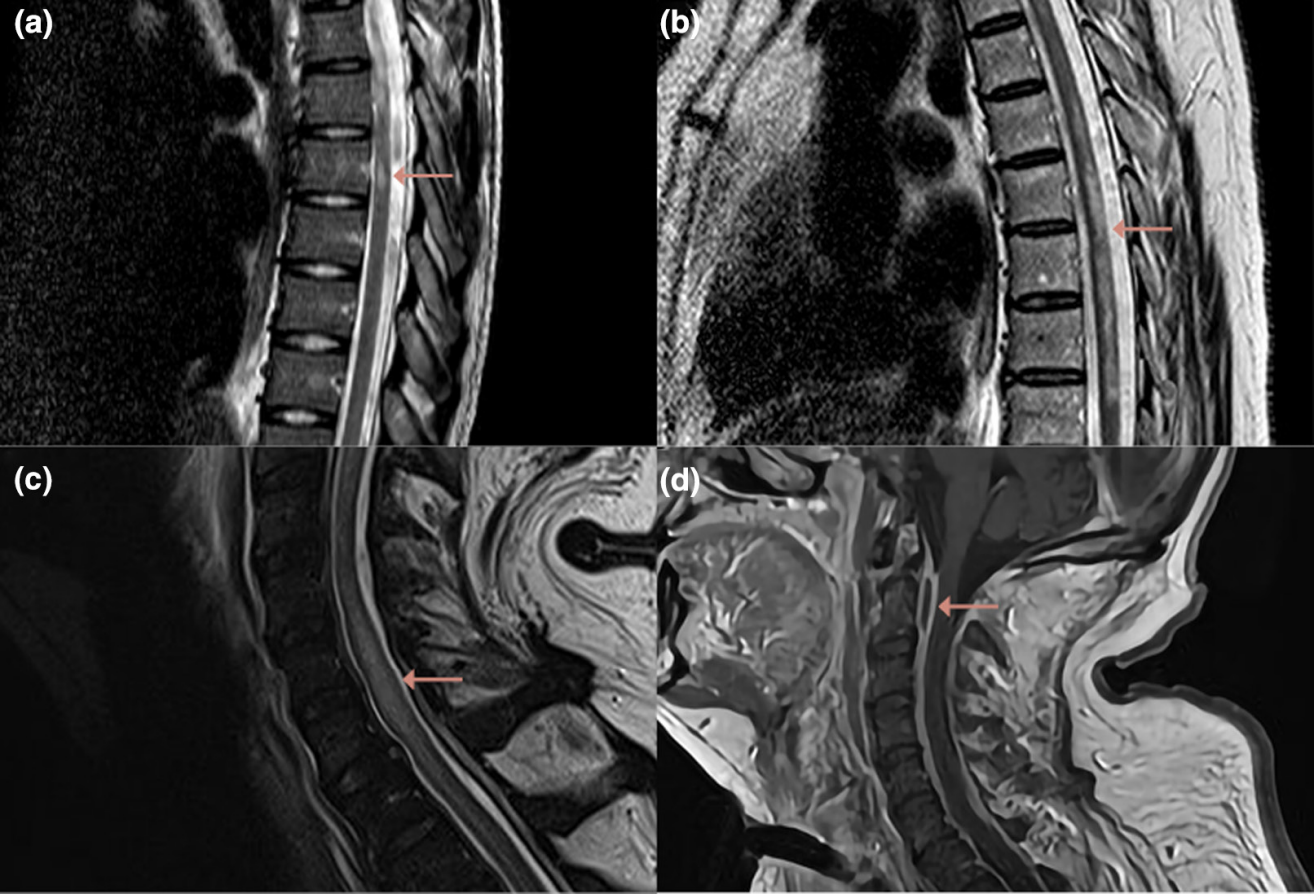
Spinal magnetic resonance imaging (MRI) images of patients with noncompressive myelopathy secondary to bacterial meningitis. (a) T2‐weighted sagittal spinal MRI shows an hyperintense lesion at T6–T7. This patient was diagnosed with postinflammatory myelitis. (b) T2‐weighted sagittal spinal MRI shows a longitudinal hyperintense lesion in a large part of the thoracic spinal cord. This patient was also diagnosed with postinflammatory myelitis. (c, d) T2‐weighted sagittal spinal MRI shows a longitudinal hyperintense lesion in the lower cervical spinal cord (c) and an epidural abscess at C1–C2 (d) on T1‐weighted images. This patient was diagnosed with infectious myelitis.

Spinal MRI revealed T2‐weighted abnormalities in six of seven patients (Figure 1). Abnormalities were seen in the thoracic spinal cord in four of six patients and in the cervical spinal cord in three of six patients. Longitudinally extensive T2 lesions, defined as lesions extending across three or more vertebral segments, were reported in three patients. Gadolinium was administered in three patients and showed spinal cord enhancement in one. One patient had multiple epidural abscesses on spinal MRI, which did not cause compression (Figure 1d). These abscesses were, therefore, not considered to be the cause of the myelopathy. Lumbar puncture was repeated in three of seven patients, revealing improvement of CSF abnormalities and negative cultures in all patients. No patients were tested for myelin oligodendrocyte glycoprotein (MOG)–IgG or aquaporin‐4 (AQP4)–IgG. Two patients were diagnosed with postinflammatory myelitis and were subsequently treated with high‐dose corticosteroids (Figure 1a,b). One patient was diagnosed with infectious myelitis and treated with penicillin for 6 weeks (Figure 1c,d). Two patients were diagnosed with spinal infarction, and in the other two patients no specific diagnosis was made; these four patients were not treated with immunosuppressive treatment.

Improvement of spinal cord function was noted in four of seven patients. All patients had an AIS grade C or higher at discharge, but only two patients attained an AIS grade D at discharge and regained functional motor ability. Bladder and bowel dysfunction persisted in all five patients. Outcome was considered unfavorable in all seven patients, because all patients were not able to live independently and were discharged to a rehabilitation center.

### Review of literature

Fourteen case reports that were relevant to our review were derived from the literature search, describing 15 adult patients with noncompressive myelopathy secondary to community‐acquired bacterial meningitis (Table S2). We combined these patients with our cases, resulting in a patient cohort of 22 adult patients, and described their clinical characteristics and outcome (Table 1).

**TABLE 1:** Clinical characteristics and outcome of 22 patients with bacterial meningitis complicated by noncompressive myelopathy.

Characteristic	All, *N* = 22
Age, years	36 (20–62)
Female sex	9/22 (41%)
Predisposing conditions	
Immunocompromised before admission	3/22 (14%)
Diabetes mellitus	2/22 (9%)
Splenectomy	1/22 (5%)
Otitis	4/22 (18%)
Endocarditis	1/22 (5%)
Symptoms on presentation	
GCS score on admission	11 (7–14)
Focal neurologic signs on admission	7/22 (32%)
Paresis	5/22 (23%)
Cranial nerve palsy	4/22 (18%)
CSF examination	
Leukocytes, cells/mm^3^	6197 (1144–29,850)
CSF protein, g/L	4.9 (2.4–6.9)
CSF glucose, mmol/L	0.8 (0.2–2.1)
Microbiology	
*Neisseria meningitidis*	9/22 (41%)
*Streptococcus pnuemoniae*	7/22 (32%)
*Staphylococcus aureus*	1/22 (5%)
*Haemophilus influenzae*	1/22 (5%)
*Streptococcus agalactiae*	2/22 (9%)
Negative culture	2/22 (9%)
Spinal cord dysfunction	
Days until symptoms	5 (1–9)
Paraplegia	16/22 (73%)
Tetraplegia	5/22 (23%)
Bladder or bowel dysfunction	18/19 (95%)
AIS grade [19]	
A–complete impairment	9/22 (41%)
B–altered sensory but no motor function	4/22 (18%)
C–minimal motor function	5/22 (23%)
D–functional motor function	4/22 (18%)
E–normal motor and sensory function	0/22 (0%)
Radiology	
MRI spine performed	19/22 (86%)
Abnormal MRI	16/19 (84%)
T2 abnormalities in cervical spine	6/16 (38%)
T2 abnormalities in thoracic spine	12/16 (75%)
Contrast enhancement	4/16 (25%)
Epidural abscess	1/16 (6%)
Treatment spinal cord injury	
Corticosteroid	8/22 (36%)
Plasmapheresis	2/22 (9%)
Antibiotics	2/22 (9%)
Outcome	
Unfavorable outcome, GOS 1–4	20/22 (91%)
Mortality	2/22 (9%)
AIS grade at discharge	
A–complete impairment	3/20 (15%)
B–altered sensory but no motor function	2/20 (10%)
C–minimal motor function	6/20 (30%)
D–functional motor function	9/20 (45%)
E–normal motor and sensory function	0/20 (0%)
Improvement in AIS grade	10/20 (50%)

*Note*: Data are shown as median [interquartile range] or *n*/*N* (%). CSF leukocytes were reported in 18 patients, CSF protein in 16 patients, and CSF glucose in 16 patients.

Abbreviations: AIS, American Spinal Injury Association Impairment Scale; CSF, cerebrospinal fluid; GCS, Glasgow Coma Scale; GOS, Glasgow Outcome Scale; MRI, magnetic resonance imaging.

Nine of 22 patients were female (41%), and the median age was 36 years (interquartile range [IQR] = 20–62; Table 1). The median Glasgow Coma Scale on admission was 11 (IQR = 7–14). Lumbar puncture was performed in all patients. CSF leukocyte count was reported in 18 cases and showed a median count of 6197 cells/mm^3^ (IQR = 1144–29,850). CSF protein level was reported in 16 patients, and the median level was 4.9 g/L (IQR = 2.4–6.9). The causative pathogen was *N. meningitidis* in nine patients (41%), *S. pneumoniae* in seven patients (32%), *S. agalactiae* in two patients (9%), and *Staphylococcus aureus* and *H. influenzae* both in one patient (5%). In two of 22 patients (9%), no causative pathogen was cultured. All patients were treated with adequate antibiotic treatment against the causative pathogen. Fourteen of 22 patients (64%) received adjunctive intravenous corticosteroids simultaneously with the initial antibiotics.

All patients developed symptoms related to spinal cord dysfunction, with a median duration of 5 days (IQR = 1–10). Sixteen of 22 patients (73%) had motor deficits of only the lower extremities and five of 22 (23%) in all four extremities. All patients had sensory changes below the level of injury. Complete spinal cord dysfunction from the site of injury, graded as AIS grade A, was observed in nine of 22 patients (41%). The remaining patients exhibited incomplete spinal cord injury, with four of 22 patients demonstrating absence of motor function but retention of some sensory function (graded as AIS grade B), five of 22 patients showing partial motor function (graded as AIS grade C), and four of 22 patients showing retention of functional motor ability (graded as grade D). Bladder or bowel dysfunction was reported in 17 of 22 patients (77%).

A spinal MRI scan was performed in 19 of 22 patients (86%) and showed abnormalities in the spinal cord on T2‐weighted images in 16 patients (84%). Spinal compression was ruled out in all patients with spinal MRI or spinal CT. MRI abnormalities were seen in the thoracic spinal cord in 12 of 16 patients (75%) and in the cervical spinal cord in six of 16 patients (38%). Lesions with contrast enhancement were observed in four of 16 patients (25%). Longitudinally, T2 lesions were present in eight of 16 patients (50%). One patient had epidural abscesses. Follow‐up spinal MRI was performed in five patients after a median interval of 11 days from the previous spinal MRI (range = 5–19 days). Complete improvement was observed in one patient, and lesion expansion was noted in three patients. The MRI abnormalities of the other patient were consistent with previous findings. Based on clinical presentation and MRI findings, six patients were diagnosed with myelitis and seven patients with spinal infarction. One patient was diagnosed with infectious myelitis. For the remaining patients, a specific diagnosis was not provided. Eight of 22 patients were additionally treated for autoimmune myelitis with high‐dose corticosteroids (36%), and two of these also received plasmapheresis. Two patients were treated with prolonged antibiotic treatment (9%).

Outcome was graded at discharge according to the GOS. Only two of 22 patients (9%) had a favorable outcome (GOS 5), and two of 22 patients (9%) died during clinical course (GOS 1). Improvement of spinal cord function during admission was noted in 10 of the 20 surviving patients (50%). At discharge, three of 20 patients still experienced complete spinal cord dysfunction from the site of injury (AIS grade A). Eight of 20 patients regained functional motor ability (AIS grade D or higher). Among the patients who received immunosuppressive therapy, five of eight patients experienced at least one grade improvement in AIS grade during admission (63%). Among those who did not receive immunosuppressive therapy, five of 14 patients showed improvement in AIS grade (36%). Nevertheless, this difference was not statistically significant (*p* = 0.44).

## DISCUSSION

Our study shows that noncompressive spinal cord dysfunction is a rare but severe complication of community‐acquired bacterial meningitis. Over a 17‐year period, we identified seven episodes in our nationwide cohort of noncompressive myelopathy secondary to bacterial meningitis. A review of literature showed an additional 15 adult patients with this complication. By combining these data, we found that this complication was not specific to one pathogen nor to any identifiable risk factors. Symptoms indicative of spinal cord dysfunction were typically manifested several days after the onset of bacterial meningitis, in some cases after an initial clinical improvement. Myelopathy was often accompanied by the development of other focal abnormalities, mainly cranial nerve palsies, indicating a severe secondary inflammatory response.

The delayed occurrence of symptoms, after initial clinical improvement in bacterial meningitis, is also seen in patients with delayed cerebral thrombosis [21]. Delayed cerebral thrombosis presents as a clinical deterioration, typically a sudden decline in consciousness, >5 days after meningitis onset. Brain imaging shows new widespread ischemic lesions. In an explorative analysis, patients with delayed cerebral thrombosis had eightfold higher complement C5a CSF concentrations on the diagnostic lumbar puncture as compared in those without delayed cerebral thrombosis [22]. Similar mechanisms may play a role in certain patients with myelopathy secondary to bacterial meningitis, where delayed spinal thrombosis may occur. However, not all patients exhibited deterioration following initial clinical improvement, indicating the involvement of other mechanisms contributing to the pathology. Additional potential etiologies of noncompressive myelopathy secondary to bacterial meningitis include other postinflammatory syndromes involving the spinal cord, such as acute disseminated encephalomyelitis or transverse myelitis, as well as direct infection of the spinal cord or spinal infarction. Spinal infarction can result from thromboembolic events due to a hypercoagulability state during infection, hemodynamic changes, or vasculitis secondary to bacterial meningitis, which is also believed to occur in delayed cerebral thrombosis [23].

Postinflammatory myelitis is often reported after the acute phase of infection, whereas infectious myelitis and spinal infarction are more likely to occur in the infectious phase. Spinal infarction is characterized by an acute onset of symptoms, whereas postinflammatory and infectious myelitis are characterized by progression of symptoms over several days [24]. Typical MRI findings of spinal infarction include a hyperintense lesion on T2‐weighted images, which is often located in the thoracic anterior spinal cord. Subsequently, restricted diffusion on diffusion‐weighted imaging (DWI) and the absence of contrast enhancement are suggestive for spinal cord infarction [24]. Typical MRI findings of postinflammatory myelitis are longitudinal lesions that often exhibit contrast enhancement and swelling [24]. The MRI findings of myelitis due to direct bacterial infection of the spinal cord are not well described in literature. A patchier pattern of hyperintense lesions is described in some cases, in which contrast enhancement and restricted diffusion in DWI images are present [25]. Abnormalities on spinal MRI were seen in most patients within our cohort, although they were frequently nonspecific, posing challenges in establishing a definitive diagnosis. Subsequently, monitoring clinical progression in these patients proves challenging because of impaired consciousness due to bacterial meningitis. It is probable that all abovementioned mechanisms contribute to the pathophysiology of noncompressive myelopathy in bacterial meningitis. An additional diagnostic clue could be the identification of antibodies associated with myelitis, such as MOG‐IgG and AQP4–IgG. However, these tests were not performed on the patients included in this study. Antibody‐mediated myelitis has been previously described following neurological varicella‐zoster infections and SARS‐CoV‐2 infections [26, 27]. Moreover, antibody‐mediated disorders are increasingly recognized following neurological infections, including anti‐N‐methyl‐D‐aspartate receptor encephalitis after herpes simplex virus encephalitis [28]. Therefore, it could be relevant to include MOG‐IgG and AQP4‐IgG in the diagnostic workup of noncompressive myelitis secondary to bacterial meningitis.

Previous research has documented the significant role of inflammation in the severity of spinal cord injuries across all etiologies [29]. It is reasonable to assume that inflammation contributes to the pathophysiology of all cases examined in this study. Therefore, administrating immunosuppressive drugs may offer therapeutic benefits. In this study, we saw a higher proportion of improvement in spinal cord function among the patients who were subjected to immunosuppressive treatment compared to the untreated group. None of the patients receiving immunosuppressive drugs exhibited deterioration of symptoms. Nevertheless, substantial uncertainty remains about the type, dose, and effectiveness of additional immunosuppressive treatment in these patients. Moreover, it is important to note that during clinical course a considerable proportion of patients exhibited some degree of improvement in spinal cord function. Nevertheless, these improvements often failed to translate into favorable outcomes.

Our study has several limitations. First, patients were only included from our nationwide cohort if they were displaying symptoms suggestive of spinal cord dysfunction and imaging was conducted to rule out spinal compression. There is a possibility that cases with a severe clinical course, resulting in rapid mortality or lack of improvement in consciousness, may be overlooked and thereby lead to an underestimation of the case number. Second, we incorporated case reports into our analysis, which are subjected to publication bias and may not accurately represent the broader population. Nevertheless, we observed that noncompressive myelopathy is a rare yet severe complication of bacterial meningitis, often resulting in devastating outcomes for affected patients. Timely recognition of spinal cord dysfunction can be challenging due to the presence of impaired consciousness secondary to bacterial meningitis. When suspicion arises, prompt spinal MRI evaluation is essential. Patients afflicted with noncompressive myelopathy potentially benefit from immunosuppressive treatment. Nevertheless, the prognosis remains unfavorable for a considerable number of patients, primarily due to neurological sequelae stemming from spinal cord dysfunction.

## AUTHOR CONTRIBUTIONS

**Evelien H. G. M. Drost:** Data curation (equal); resources (equal); writing–original draft (lead); writing–review and editing (equal); formal analysis (lead). **Nora Chekrouni:** Data curation (equal); resources (equal). **Matthijs C. Brouwer:** Conceptualization (equal); writing–review and editing (equal). **Diederik van de Beek:** Conceptualization (equal); writing–review and editing (equal).

## FUNDING INFORMATION

The work was supported by the Netherlands Organization for Health Research and Development (ZonMw; NWO‐Vidi‐Grant [grant number 917.17.308] to M.C.B.; NWO‐Vici‐Grant [grant number 918.19.627] to D.v.d.B.).

## CONFLICT OF INTEREST STATEMENT

The authors report no competing interests.

## Supporting information


Tables S1–S2.

## Data Availability

Data protection regulations in the Netherlands do not allow sharing of individual participant data. Datasets with selected aggregated data will be shared upon request. Proposals can be directed to meningitis@amsterdamumc.nl.
