# Supplementary material for: Noncompressive myelopathy in acute community‐acquired bacterial meningitis: Report of seven cases and review of literature
Source: Eur J Neurol. 2024 Sep 2;31(12):e16447. doi: 10.1111/ene.16447 (PMC11555019; doi:10.1111/ene.16447)
Supplement: Supplementary file 1 — Tables S1–S2. [file ENE-31-e16447-s001.docx]

| **Table 1 Clinical characteristics and outcome of 22 patients with bacterial meningitis complicated by non-compressive myelopathy** | |
| --- | --- |
| **Characteristic** | **All, N = 22** |
| Age – years | 36 (20 – 62) |
| Female sex | 9/22 (41%) |
| **Predisposing conditions** |  |
| Immunocompromised before admission | 3/22 (14%) |
| Diabetes mellitus | 2/22 (9%) |
| Splenectomy | 1/22 (5%) |
| Otitis | 4/22 (18%) |
| Endocarditis | 1/22 (5%) |
| **Symptoms on presentation** |  |
| GCS score on admission | 11 (7-14) |
| Focal neurologic signs on admission | 7/22 (32%) |
| Paresis | 5/22 (23%) |
| Cranial nerve palsy | 4/22 (18%) |
| **CSF examination** |  |
| Leukocytes - cells/mm3 | 6,197 (1,144 – 29,850) |
| CSF protein - g/L | 4.9 (2.4 – 6.9) |
| CSF glucose - mmol/L | 0.8 (0.2 – 2.1) |
| **Microbiology** |  |
| *Neisseria meningitidis* | 9/22 (41%) |
| *Streptococcus pnuemoniae* | 7/22 (32%) |
| *Staphylococcus aureus* | 1/22 (5%) |
| *Haemophilus influenzae* | 1/22 (5%) |
| *Streptococcus agalactiae* | 2/22 (9%) |
| Negative culture | 2/22 (9%) |
| **Spinal cord dysfunction** |  |
| Days till symptoms | 5 (1-9) |
| Paraplegia | 16/22 (73%) |
| Tetraplegia | 5/22 (23%) |
| Bladder or bowel dysfunction | 18/19 (95%) |
| AIS grade † |  |
| A – complete impairment | 9/22 (41%) |
| B – altered sensory but no motor function | 4/22 (18%) |
| C – minimal motor function | 5/22 (23%) |
| D – functional motor function | 4/22 (18%) |
| E – normal motor and sensory function | 0/22 (0%) |
| **Radiology** |  |
| MRI spine performed | 19/22 (86%) |
| Abnormal MRI | 16/19 (84%) |
| T2 abnormalities cervical spine | 6/16 (38%) |
| T2 abnormalities thoracic spine | 12/16 (75%) |
| Contrast enhancement | 4/16 (25%) |
| Epidural abscess | 1/16 (6%) |
| **Treatment spinal cord injury** |  |
| Corticosteroid | 8/22 (36%) |
| Plasmapheresis | 2/22 (9%) |
| Antibiotics | 2/22 (9%) |
| **Outcome** |  |
| Unfavourable outcome (GOS 1-4) | 20/22 (91%) |
| Mortality | 2/22 (9%) |
| AIS^1^ grade at discharge |  |
| A – complete impairment | 3/20 (15%) |
| B – altered sensory but no motor function | 2/20 (10%) |
| C – minimal motor function | 6/20 (30%) |
| D – functional motor function | 9/20 (45%) |
| E – normal motor and sensory function | 0/20 (0%) |
| Improvement in AIS^1^ grade | 10/20 (50%) |
| † American Spinal Injury Association Impairment Scale ^19^ Data are shown as median [IQR] or n/N (%). CSF leukocytes was reported in 18 patients, CSF protein in 16 patients, CSF glucose in 16 patients. Abbreviations: GCS=Glasgow Coma Scale, CSF=cerebrospinal fluid, MRI = magnetic resonance imaging, GOS = Glasgow Outcome Scale. | |

**FIGURE LEGENDS**

**Figure 1 Spinal MRI images of patients with non-compressive myelopathy secondary to bacterial meningitis.** (**A**) T2-weighted sagittal spinal MRI shows an hyperintense lesion at T6-T7. This patient was diagnosed as post-inflammatory myelitis. (**B**) T2-weighted sagittal spinal MRI shows a longitudinal hyperintense lesion in a large part of the thoracic spinal cord. This patient was also diagnosed as post-inflammatory myelitis. (**C**) T2-weighted sagittal spinal MRI shows a longitudinal hyperintense lesion in the lower cervical spinal cord and an epidural abscess (**D**) at C1-C2 on T1-weighted images. This patient was diagnosed with infectious myelitis.

**SUPPLEMENTARY TABLES**

| **Supplementary Table 1 Clinical characteristics of 7 patients with non-compressive myelopathy secondary to bacterial meningitis** | | | | | | | |
| --- | --- | --- | --- | --- | --- | --- | --- |
|  | **Patient 1** | **Patient 2** | **Patient 3** | **Patient 4** | **Patient 5** | **Patient 6** | **Patient 7** |
| **Immunocompromised before admission** | No | No | No | No | No | No | No |
| **Predisposing factor** | None | None | Endocarditis | Sinusitis | Otitis, sinusitis | None | Otitis |
| **Symptoms at admission** |  |  |  |  |  |  |  |
| **Temperature (℃)** | 38.8 | 38.8 | 40.3 | 34.2 | 37.2 | 39.5 | 37.2 |
| **Neck stiffness** | Yes | No | Yes | Yes | No | Yes | Yes |
| **Score on Glasgow Coma Scale** | 7 | 7 | 10 | 8 | 14 | 13 | 10 |
| **Focal neurological deficits on admission** | None | None | Right sided hemiparesis and aphasia | N3 palsy | None | None | None |
| **Computed tomography (CT) on admission** | Not performed | No abnormalities | No abnormalities | Sinus opacification | Mastoid and sinus opacification | No abnormalities | Mastoid opacification |
| **CSF examination** |  |  |  |  |  |  |  |
| **Leukocytes – cells/mm^3^** | 43200 | Not performed | 55 | 2200 | 2987 | 4580 | 7814 |
| **Protein – g/L** | 6.7 | Not performed | 0.67 | 3.4 | 6.4 | 9.7 | 9.9 |
| **Glucose – mmol/L** | 0.1 | Not performed | 3.40 | 0.10 | 0.1 | 9.1 | 0.2 |
| **Culture** | *N. meningitidis* | *S. pneumoniae* | *S. agalactiae* | *S. pneumoniae* | *H. Influenzae* | *S. agalactiae* | *S. pneumoniae* |
| **Initial antibiotic therapy** | Cefotaxime | Penicillin | Ceftriaxone | Ceftriaxone | Ceftriaxone, amoxicillin | Ceftriaxone, amoxicillin | Ceftriaxone, amoxicillin |
| **Adjunctive dexamethasone** | Yes | No | Yes | Yes | Yes | Yes | Yes |
| **Time till spinal cord dysfunction (days)** | 2 | 9 | 5 | 7 | 13 | 14 | 28 |
| **Neurological deficits due to spinal cord dysfunction** | Paresis lower extremities. | Paralysis lower extremities. | Paresis lower extremities. | Paresis lower extremities, sensory changes below Th8. | Paresis lower extremities, sensory changes below Th7 | Progressive tetraparesis | Paresis lower extremities. |
| **AIS^1^ grade** | C | B | D | C | C | B | A |
| **Spinal MRI** | Multiple T2-hyperintense lesions at T6-T7 and T3-T4. Contrast series not performed. | T2 -hyperintense lesion in the entire thoracic spinal cord with swelling. Contrast series not performed. | T2-hyperintense lesion in the cervical spinal cord. Contrast series not performed | No compression, no abnormalities in the spinal cord | Longitudinal T2-hyperintense lesion in the entire thoracic spinal cord, with contrast enhancement | Longitudinal T2-hyperintense lesion mostly spinal cord. No contrast enhancement. Small abscesses in cervical and lumbar spinal cord | Multiple T2-hyperintense lesions in the cervical and thoracic spinal cord. No contrast enhancement. |
| **Treatment** | High dose dexamethasone (dose not reported) | None | None | None | Methylprednisolone 1000 mg for 3 days | Penicillin for 6 weeks | None |
| **Other neurological deficits** | Right-sided N6 palsy | Left-sided N3 palsy. Ptosis both eyes. | Right-sided hemiparesis and aphasia | Right-sided N7 palsy | None | None | Right-sided N7 and N3 palsy. |
| **Other complications during admission** | Pneumonia | Circulatory shock, erysipelas | Cerebral infarction, pneumonia | Epilepsy | Pneumonia | Pneumonia, polymyalgia rheumatica | Epilepsy, hydrocephalus, cerebral infarction, circulatory shock |
| **Clinical outcome** | Moderately disabled | Severely disabled | Severely disabled | Moderately disabled | Moderately disabled | Severely disabled | Severely disabled |
| **Neurological deficits at discharge** | Improving paresis lower extremities, N6 palsy | Paresis lower extremities, N3 palsy | Paresis lower extremities. Right sided hemiparesis and aphasia | Paresis lower extremities, sensory changes below Th8. N7 palsy | Paresis lower extremities | Tetraparesis | Paresis lower extremities. N7 and N3 palsy. |
| **AIS^1^ grade at discharge** | D | C | D | C | C | C | C |
| **Final diagnosis** | Post-inflammatory myelitis | No specific diagnosis | Spinal infarction | No specific diagnosis | Post-inflammatory myelitis | Infectious myelitis | Spinal infarction |
| ^1.^American Spinal Injury Association Impairment Scale, see Table 1 ^19^ | | | | | |  |  |

**Supplementary Table 2 Included articles in literature review**

| **First author (reference)** | **Country** | **Publication year** | **Number of patients described** |
| --- | --- | --- | --- |
| Kastenbauer et al.^4^ | Germany | 2001 | 2 |
| Ng et al. ^13^ | Malaysia | 2018 | 1 |
| De Schryver et al. ^12^ | Belgium | 2011 | 1 |
| Rathore et al. ^14^ | Pakistan | 2008 | 1 |
| Williams et al. ^15^ | United Kingdom | 2012 | 1 |
| Titlic et al. ^9^ | Croatia | 2006 | 1 |
| O’Farrell et al. ^8^ | Ireland | 2000 | 1 |
| Boothman et al. ^11^ | United Kingdom | 1988 | 1 |
| Bhojo et al. ^6^ | Pakistan | 2001 | 1 |
| Choudhary et al. | India | 2007 | 1 |
| Viskovic et al. ^10^ | Croatia | 2014 | 1 |
| Abdallah et al. ^5^ | United States | 2020 | 1 |
| Khan et al. ^7^ | Pakistan | 1990 | 1 |
| Kato et al. ^16^ | Japan | 2015 | 1 |
